# Neurobiological mechanisms of ECT and TMS treatment in depression: study protocol of a multimodal magnetic resonance investigation

**DOI:** 10.1186/s12888-023-05239-0

**Published:** 2023-10-30

**Authors:** Leila Marie Frid, Ute Kessler, Olga Therese Ousdal, Åsa Hammar, Jan Haavik, Frank Riemer, Marco Hirnstein, Lars Ersland, Vera Jane Erchinger, Eivind Haga Ronold, Gyrid Nygaard, Petter Jakobsen, Alexander R. Craven, Berge Osnes, Renata Alisauskiene, Hauke Bartsch, Stephanie Le Hellard, Anne-Kristin Stavrum, Ketil J. Oedegaard, Leif Oltedal

**Affiliations:** 1 Mohn Medical Imaging and Visualization Centre, Department of Radiology, Haukeland University Hospital, Bergen, Norway; 2 Department of Clinical Medicine, University of Bergen, Bergen, Norway; 3 Division of Psychiatry, Haukeland University Hospital, Bergen, Norway; 4 Department of Biomedicine, University of Bergen, Bergen, Norway; 5 Department of Biological and Medical Psychology, University of Bergen, Bergen, Norway; 6 Department of Clinical Sciences Lund, Psychiatry, Faculty of Medicine, Lund University, Lund, Sweden; 7 Office for Psychiatry and Habilitation, , Psychiatry Research Skåne, Region Skåne, Sweden; 8 Department of Clinical Engineering, Haukeland University Hospital, Bergen, Norway; 9 Department of Neurology, Haukeland University Hospital, Bergen, Norway; 10 NORMENT, Division of Psychiatry, Haukeland University Hospital, Bergen, Norway; 11 Department of Clinical Psychology, University of Bergen, Bergen, Norway; 12 NORMENT, Department of Clinical Science, University of Bergen, Bergen, Norway; 13 Dr. Einar Martens Research Group for Biological Psychiatry, Department of Medical Genetics, Haukeland University Hospital, Bergen, Norway

**Keywords:** Electroconvulsive therapy, Magnetic resonance imaging, Transcranial magnetic stimulation, Major depressive disorder, Cognitive deficits

## Abstract

**Background:**

Noninvasive neurostimulation treatments are increasingly being used to treat major depression, which is a common cause of disability worldwide. While electroconvulsive therapy (ECT) and transcranial magnetic stimulation (TMS) are both effective in treating depressive episodes, their mechanisms of action are, however, not completely understood. ECT is given under general anesthesia, where an electrical pulse is administered through electrodes placed on the patient’s head to trigger a seizure. ECT is used for the most severe cases of depression and is usually not prescribed before other options have failed. With TMS, brain stimulation is achieved through rapidly changing magnetic fields that induce electric currents underneath a ferromagnetic coil. Its efficacy in depressive episodes has been well documented. This project aims to identify the neurobiological underpinnings of both the effects and side effects of the neurostimulation techniques ECT and TMS.

**Methods:**

The study will utilize a pre-post case control longitudinal design. The sample will consist of 150 subjects: 100 patients (bipolar and major depressive disorder) who are treated with either ECT (*N* = 50) or TMS (*N* = 50) and matched healthy controls (*N* = 50) not receiving any treatment. All participants will undergo multimodal magnetic resonance imaging (MRI) as well as neuropsychological and clinical assessments at multiple time points before, during and after treatment. Arterial spin labeling MRI at baseline will be used to test whether brain perfusion can predict outcomes. Signs of brain disruption, potentiation and rewiring will be explored with resting-state functional MRI, magnetic resonance spectroscopy and multishell diffusion weighted imaging (DWI). Clinical outcome will be measured by clinician assessed and patient reported outcome measures. Memory-related side effects will be investigated, and specific tests of spatial navigation to test hippocampal function will be administered both before and after treatment. Blood samples will be stored in a biobank for future analyses. The observation time is 6 months. Data will be explored in light of the recently proposed *disrupt, potentiate and rewire* (DPR) hypothesis.

**Discussion:**

The study will contribute data and novel analyses important for our understanding of neurostimulation as well as for the development of enhanced and more personalized treatment.

**Trial registration:**

ClinicalTrials.gov Identifier: NCT05135897.

## Background

Major unipolar depressive disorder (MDD) and bipolar depressive disorder (BDD) are common and serious medical conditions that may lead to profound suffering, reduced quality of life, inability to work and increased risk of mortality (https://www.who.int/news-room/fact-sheets/detail/depression). These disorders are among the leading causes of disability in the world [1–3]. According to the World Health Organization, approximately 280 million people are living with depression worldwide, and the estimated number of annual global suicides is 700,000 (https://www.who.int/news-room/fact-sheets/detail/depression). The European 12-month prevalence is 1 and 7% for BDD and MDD, respectively [4], and the annual cost of mood disorders in Europe was estimated to be 113.4 Bn Euro [5]. The management of depression includes psychosocial, cognitive and pharmacotherapeutic approaches. However, approximately 30% of patients do not respond to standard treatments even after multiple treatment steps [6].

Cognitive deficits are common in depression [7–9], and the occurrence of these impairments has been described within the “*state”, “scar” and “trait”* hypotheses [10, 11]. It is still unclear whether the impairments preexist as a “*trait”* prior to MDD, whether they occur as a “*state”* during a depressive episode or whether impairments are caused by depression (“*scar”*). The *scar* hypothesis suggests that depression is neurotoxic to the brain and will cause cognitive impairment over time, consistent with studies suggesting that cognitive impairments are related to the duration and number of depressive episodes [7, 12, 13]. The “*trait”* hypothesis suggests that an underlying vulnerability prior to the start of depression can contribute to the development of symptoms in depression as well as contribute to treatment resistance and relapses [11].

### Electroconvulsive therapy (ECT)

*ECT* is a medical treatment in which electric currents delivered through scalp electrodes are used to intentionally trigger a brief seizure. Patients are under general anesthesia and muscular blockade during treatment sessions, which are provided two or three days per week for two to four weeks. ECT has remained the most effective acute treatment for major depressive episodes [14, 15] over the past 80 years. Even for patients with treatment-resistant, severe and sometimes life-threatening depressive episodes, the remission rates of ECT are up to 75% [16]. However, the use of ECT is still debated [17, 18], and the lack of an overarching model for the mechanism of action of ECT possibly contributes to its public controversy [19]. Considering the large number of people suffering from depression, the use of ECT is variable, with reported treated person rates (TPR; number of persons treated with ECT per 10,000 resident population per year) between 0.11 and 5.1 worldwide [20], possible due to worries about side effects.

ECT is associated with cognitive side effects [21, 22]. However, the literature is divergent and inconclusive regarding the nature and development of the neuropsychological profile of cognitive side effects [23, 24]. Most of the cognitive impairments seem to be limited to the first three days after ECT [25], and some patients even report an improvement in neurocognitive function [23]. While reduced autobiographical memory consistency has been well documented [26–28], a number of other cognitive functions has been shown to improve one month after ECT, compared with pre-ECT [24]. Although several guidelines highlight the importance of assessing cognitive function during ECT, there is no consensus regarding the optimal test battery and study design [24, 29], thus large well-designed studies investigating broad measures of cognition pre-post ECT are warranted.

There is substantial evidence for ECT-induced volumetric changes in brain gray matter areas [30, 31], which depend on electrode placement [32] and correlate with the electrical field strength [33]. Although there are conflicting results regarding the clinical relevance of these changes, some association with clinical response has been found [34, 35], and side effects may relate to hippocampal volume change [36, 37].

### Transcranial magnetic stimulation (TMS)

TMS is a form of brain stimulation in which rapidly changing magnetic fields induce electric currents underneath a ferromagnetic coil [38]. The stimulation comes in the form of pulses and depending on the frequency of these pulses, TMS can have excitatory or inhibitory effects in cortical regions [39]. TMS is a relatively new treatment and was approved by the US Food and Drug Administration for medication resistant depression in 2008. Based on consistent findings that show hypoactivation of the left dorsolateral prefrontal cortex (DLPFC) in MDD [40, 41], a high-frequency TMS protocol was developed to re-establish normal activity levels within this region [42], to reduce symptoms and potentially improve aspects of neurocognition [43] not currently improved by traditional treatments. The treatment typically requires daily TMS sessions of up to 3000 pulses for four to six weeks [44]. The efficacy of TMS treatment has been demonstrated in several meta-analyses as well as single- and multisite studies [44, 45]. Approximately, two-thirds of patients respond to the treatment and show some improvement, while the remission rate is approximately 30% [46].

## Methods

### Aim of the study

This project aims to improve our understanding of the fundamental, neurobiological underpinnings of the response and side effects of ECT and TMS therapy using multiple biomarkers, including genetic and biochemical markers, brain magnetic resonance imaging (MRI), actigraphy, and novel measures of spatial navigation included in the neurocognitive test battery. The long-term goal of the study is to develop an *outcome expectancy score*—a personalized probability of a good outcome.

#### Study design

**Fig. 1:**
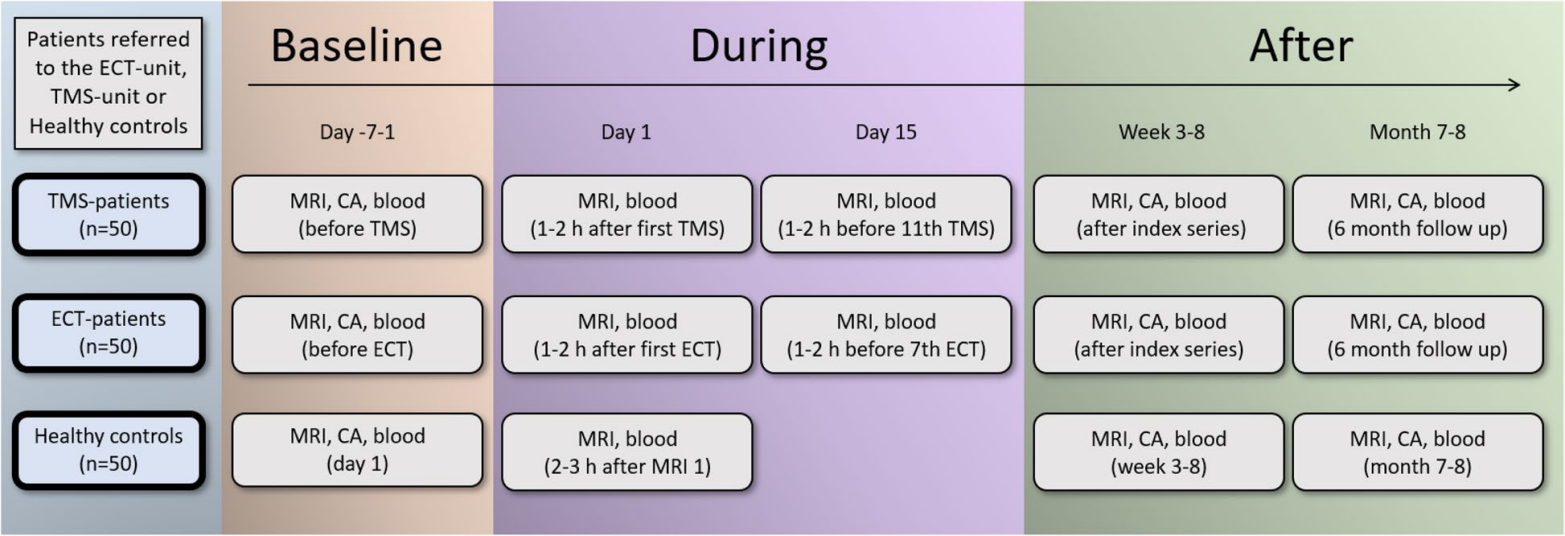
Study flow-chart. Assessments are performed at multiple time points before, during and after treatment as illustrated. CA, Core Assessment (includes neurocognitive tests, actigraphy and for the patient groups clinical assessment)

The study is a prospective pre-post longitudinal case–control observational and “naturalistic” study, i.e., we will collect data from patients who receive the standard clinical treatment and compare three groups: patients receiving ECT, patients receiving TMS, and healthy controls who do not receive any treatment (Fig. 1). The data collection is broad and comprehensive. This is necessary due to the lack of knowledge in the field; exploratory and hypothesis-generating research is important and warranted.

MRI, blood samples, neuropsychological assessments (including spatial navigation – a function residing in the hippocampus), clinical scales and depression scores from 100 patients in a major depressive episode will be collected. The ECT (*N* = 50) and TMS (*N* = 50) study groups will be followed over 5 time points (TP): baseline (TP1), during (after 1^st^ (TP2) and 6^th^ ECT or 10^th^ TMS (TP3)), after treatment series (TP4), and at 6 months follow-up (TP5). Healthy controls (*N* = 50) will be assessed at four time points (corresponding to TP1, TP2, TP4 and TP5 to estimate variance due to noise, and to control for effects of time. There are comprehensive and in-depth assessments of participants, spanning from diagnosis, history and imaging to neuropsychological profiling, blood biomarkers, genetics, and epigenetics (see Fig. 1 and Table 1).

**Table 1:** Variable overview

	Timepoints (TP) for analyses	TP1		TP2	TP3	TP4	TP5
	Timepoints relative to treatment	Baseline, before treatment		During treatment		After treatment	
	Timeframe for each test	< 7 d of first ECT/TMS	1–2 h before first ECT/TMS	1–2 h after first ECT/TMS	before 7th ECT/11th TMS	7–14 d after last ECT/TMS	6 mo after ECT/TMS series
Clinical assessments ^c^	Clinical examination	x					
ECT- stimulus specific measures ^b^	EEG, pulse width, charge, amplitude, frequency, pulse train duration, seizure duration, electrode placement and reorientation time		x		x		
	Current and concomitant medication	x			x	x	x
Symptom severity ^c^	MADRS [47]	x			x	x	x
	BDI [48]	x			x	x	x
	CGI [49]	x			x	x	x
	EMQ [50]	x			x	x	x
	MMSE [51]						
Radiology	MRI: T1, T2, MRS, ASL, DWI, rsfMRI		x	x	x	x	x
Neurocognitive assessments aligned with GEMRIC recommendation	1. RAVLT [52, 53]	x				x	x
	2. Phonemic Fluency [54]	x				x	x
	3. Semantic Fluency [54]	x				x	x
	4. SDMT [55]	x				x	x
	5. TMT A + B [54]	x				x	x
	7. DSB [55]	x				x	x
	8. AMT [56]	x				x	x
	9. MoCa version 7.1 [57]	x				x	x
	10. NART [58]	x				x	x
	11. QIDS-SR 16 [59]	x				x	x
Study specific neurocognitive assessments	1.VAMS^a^,^c^ [60]	x				x	x
	3. Spatial navigation: Memory retrieval task [61, 62], Four Mountain [63], Sea Hero [64]	x				x	x
	4. RCFT [65]	x				x	x
	5. CWIT [54]	x				x	x
	6. PDQ-5 [66, 67] ^c^	x				x	x
	7. ECCA [68] ^c^	x				x	x
Brain laterality, cognitive control	iDichotic [69, 70]	x				x	x
Blood samples	Serum, EDTA, PAX-gen		x		x	x	x
Actigraphy	Empatica 4 [71] ^b^	x		x	x	x	x

*Abbreviations: TP* Timepoint, *MADRS* Montgomery Åsberg Depression Rating Scale, *BDI* Beck Inventory test, *CGI* Global Impression Scale, *EMQ* Everyday Memory Questionary, *MMSE* Mini Mental Status, *RAVLT* Rey Auditory Verbal Learning Test, *F-A-S* Phonemic Fluency, *SDMT* Symbol Digit Modality Test, *TMT* Trail Making Test, *DSB* Digit Span Backwards, *AMT* Autobiographical Memory Test, *NART* National Adult Reading Test, *QIDS-SR 16* Quick Inventory of Depressive Symptomatology (16-item) (Self-Report), *VAMS* Visual Analog Mood Scale, *RCFT* Rey-Osterrieth Complex Figure Test, *CWIT* Color Word Interference Test, *EEG* electroencephalografy, *PDQ-5* Perceived Deficits Questionary – Depression 5-item, *ECCA* Electroconvulsive Cognitive Assessment, *iDichotic* Dichotic listening, *EDTA* Ethylene Diamine-Tetra-acetic Acid, *PAX-gen* PAXgene Blood RNA tube, *Empatica 4* Actigraphy with a wristband; *MRS* Magnetic Resonance Spectroscopy, *ASL* Arterial Spin Labeling, *DWI* Diffusion Weighted Imaging, *rsfMRI* Resting state functional magnetic resonance imaging

^a^Repeated before and after each treatment

^b^Repeated at each ECT treatment, TMS patients follow the same timepoints as the ECT patients

^c^Will not be assessed for healthy controls

### Recruitment and eligibility criteria

Patients are recruited when referred to ECT or TMS at Haukeland University Hospital, and healthy controls are recruited in the same geographical region as the patients. Both patients and healthy controls must understand and sign the informed consent form. All study participants receive a universal gift card to 200 Norwegian kroner (NOK) for each MRI and 300 NOK for each neuropsychological assessment in the study.

#### Inclusion criteria

*ECT/TMS:* Patients (age > 18 years) referred to ECT or TMS and accepted for treatment because of moderate and severe depression, fulfilling the criteria for the following ICD-10 diagnoses: F31.3 and F31.4; F32.1 and F32.2 and F32.3; F33.1 and F33.2 and F33.3. In addition, the symptom intensity must be verified by a Montgomery and Aasberg Depression Rating Scale (MADRS) [47] score ≥ 25.

#### Exclusion criteria

ECT/TMS treatment within the last 12 months. Patients unable to give informed consent (according to the responsible ECT/TMS clinician). Patients who cannot participate in the MR scanning due to contraindications to MRI.

#### Healthy controls

Age and sex, matched to the ECT patient group, with no history of mental disorder. There is no contraindication for MRI scanning. Healthy controls will also be screened for medical conditions and drug use.

### ECT

ECT will be administered with right unilateral (RUL) electrode positioning [72]. On clinical indication, electrode positioning can be switched to bilateral positioning (BL). The ECT device used is a Thymatron System IV (Somatic Inc.) that provides a brief pulse, square wave, and constant current (900 mA). The initial stimulus dose will be determined by an age-based method as described in Kessler et al. 2010 [73]. The initial duration of the stimulus pulse will be set to 0.5 ms. Treatment frequency is thrice weekly until remission or until no further improvement is expected, with an upper limit of 18 sessions. Each seizure will be evaluated on the following variables: duration, δ-waves, reorientation time and improvement in depression. The stimulus parameters can be adjusted in each session, based on evaluations of previous sessions.

#### Anesthesia

The anesthetic agent used for induction to ECT will be either propofol, etomidate or thiopental, as decided by the anesthesiologist. All patients are preoxygenated one to two minutes before treatment with oxygen-enriched air. All patients will also receive the muscle relaxant suxamethonium about 90 s before the stimulus is given. Hyperventilation should be maintained during this period even with high oxygen saturation. The use of other medication deemed necessary during anesthesia will be decided by the anesthesiologist.

### TMS

Treatment is delivered with a TMS stimulator (MAG & More GmbH, München, Germany) using a figure-of-eight stimulation coil (Fo8 coil). After determination of the motor threshold at the first session, (defined at the point of maximal stimulation for the right abductor pollicis brevis or other hand muscles, as visually detected, with the paddle axis oriented laterally) the coil is placed over a point 5 cm anterior to the point at which the motor threshold is obtained. Each patient will receive 5 treatment sessions per week for 6 weeks (a total of 30 sessions). Each session comprises a protocol of 1500 TMS pulses in total, delivered in 15 trains of 100 pulses per train, with 30 s intervals at a frequency of 10 Hz, at 100% of motor threshold on the treatment site over the left DLPFC. This corresponds to 9 min and 30 s stimulation in total per session.

### Clinical assessments

The clinical assessment will be performed in accordance with standard clinical protocols at the treatment unit. This includes electroencephalography (EEG), ECT stimulus specific variables (e.g., pulse width, charge delivered, amplitude, frequency, pulse train duration, seizure duration and electrode placement), reorientation time, and anesthetic used for induction. Weekly clinical assessments will include the MADRS [47], Beck Inventory test (BDI) [48], Clinical Global Impression Scale (CGI) [49], Mini Mental Status (MMSE) [51] and Everyday Memory Questionary (EMQ) [50]. Psychiatric history and the cause for cessation of ECT will be recorded. Response to treatment will be defined as > 50% reduction in MADRS score, and remission as a MADRS score ≤ 10.

### Radiological assessments

We will follow a comprehensive MRI protocol which is in accordance with recommendations from the Global ECT-MRI Research Collaboration (GEMRIC) and closely follows the standards of the Adolescent Brain Cognitive Development (ABCD) study [74, 75]. This includes various imaging sequences which will allow investigation of brain structure, brain function and metabolic profiles. This protocol includes a high resolution T1 and T2 structural volumes (sagittal 3D MPRAGE T1 with isotropic voxel size of 1 mm^3^, echo time (TE) = 3.1 ms, repetition time (TR) = 7.4 ms, flip angel (FA) = 8 deg., time of acquisition (TA) = 6:32 min, and sagittal 3D CUBE T2 with isotropic voxel size of 1 mm^3^, TE = 60 ms, TR = 3200 ms, echo train length = 140, TA = 4:46 min respectively), MR spectroscopy acquired from the right amygdala (20 × 20 × 20 mm single voxel point resolved spectroscopy, SV-PRESS, TE = 35 ms, TR = 1500 ms, 128 scans of 4096 samples, spectral width = 5000 Hz, TA = 3:48 min; and a semi-LASER (PROBE-sl) single voxel 20 × 20 × 20 mm, TE = 30 ms, TR = 2000 ms, VAPOR FA = 65, 64 scans, TA = 2:28), Arterial Spin Labeling (ASL) MRI (3D ASL with effective resolution 3.64 mm, TE = 10.5 ms, TR = 4888 ms, TA = 4:39 min), resting state fMRI (rs-fMRI using 2D echo planar imaging (EPI) with isotropic voxel size of 2.4 mm^3^, TE = 30 ms, TR = 800 ms, TA = 10 min), and multishell diffusion weighted imaging (DWI) (1.7 mm isotropic multiband-accelerated DWI with a total of 186 diffusion encoding directions measured across four non-zero b-values (6 at b = 500, 60 at b = 1000, 60 at b = 2000, 60 at b = 3000), TA 13:49 min). High Order Shim covering the whole brain will be used for the resting state magnetic resonance imaging (rsfMRI) and diffusion tensor imaging acquisitions, and EPI field maps will be generated for each. For magnetic resonance spectroscopy (MRS), we used a semi-LASER (PROBE-sl) single voxel 20 × 20 × 20 mm, TE = 30 ms, TR = 2000 ms, VAPOR flip angle = 65, 64 scans, TA = 2:28.

### Neuropsychological assessments

A test battery with standardized and normalized neuropsychological tests will be applied to investigate aspects of cognitive functioning and possible side effects of treatment, including premorbid general abilities, cognitive status, spatial navigation, language learning, verbal- visuospatial and autobiographical memory, working memory, processing speed, attention, executive functions and inhibitory control, self-perceived cognitive difficulties, and psychomotor tempo. Based on clinical observations of spatial navigation complaints after ECT, together with the structural changes in the hippocampus [32, 76], we have choosen to include specific tests of this domain as well. The complete battery has a duration of 3–4 h and will be administered by healthcare personnel, trained in the method and under supervision of a clinical neuropsychologist.

The neurocognitive test battery is aligned with the recommendations for GEMRIC [77] with the following tests: the Rey Auditory Verbal Learning Test (RAVLT) [52] where trial 1.-7. is included and delayed recall at 30 min [53]; Phonemic Fluency (F-A-S) with 60 s of naming words on each letter, Semantic Fluency (Animals + Boy’s names) with 60 s of naming words in each category, and Trail Making Test Part A and B (TMT A + B) [54] with seconds to complete and numbers of errors; Symbol Digit Modality Test (SDMT) with total numbers of correct symbols on 120 s and Digit Span Backward [55] with a total of 8 digits; Autobiographical Memory Test (AMT) [56, 78] with total numbers of specific and categorical memories retrieved within 60 min, and average seconds memory retrieval; MoCa version 7.1 [57]; National Adult Reading Test (NART) [58] and Quick Inventory of Depressive Symptomatology (16-item) (Self-Report) (QIDS-SR 16) [59]. In addition to this, the following study will include specific neurocognitive- and clinical tests: Visual Analog Mood Scale (VAMS) [60] with a scale from 0–100 performed with a 1-item question: “How depressed do you feel right now?”, which will be assessed before and after each TMS/ECT-treatment; Spatial memory navigation tests: Memory retrieval task [61, 62], Four Mountain [63] and Sea Hero [64], which is further described below; Rey—Osterrieth complex figure test (RCFT) [65] assessed with copy and delayed retrieval; D-KEFS Color Word Interference Test (CWIT) [54] included color reading, color recognition, inhibition, and inhibition-switching, allowing for a separation of improvements in processing speed and executive functions respectively [79]; Perceived Deficits Questionary – Depression (PDQ-D) 5-item [66, 67] and Electroconvulsive Cognitive Assessment (ECCA) [68].

To assess laterality effects, we adopt a dichotic listening paradigm, a well-established method to determine language specialization [69]. In dichotic listening, two different syllables are presented simultaneously to the left and right ear (e.g., “ba” to the left and “ta” to the right ear). Participants tended to report the right ear stimulus because of stronger contralateral connections with the language-specialized left hemisphere. The present study will employ a dichotic listening paradigm implemented as a smartphone app [80].

### Spatial navigation

Three tasks for assessing *spatial navigation* will be used. Although spatial navigation is a function known to be located in the hippocampal region [81], there is a lack of studies investigating spatial navigation in relation to ECT.

*The memory retrieval task* [61, 62] is performed on a computer screen, where the participant is moving in a virtual grassy plane surrounded by a circular wall. Behind the wall, the scenery contains landmarks such as mountains, the sun, and clouds. Objects appear at certain spots, and the participant uses the keyboard to navigate around the plane and pick up the objects. The task is to remember the location and replace the objects back to the same location.

*The Four Mountains test* [63] is administered using a tablet computer. First, a picture of a four-mountain landscape shows up. After a brief delay, four similar pictures appear, one of which is the same landscape as the first one but viewed under different conditions (such as a change in viewpoint or season). The task is to choose the correct landscape matching the first shown. The test includes 15 trials with different mountain landscapes.

*Sea Hero Quest* is an application from the lab of Hugo Spiers, University College London [64]. This test is downloadable to a handheld device, and for the current study, we use a tablet computer. The task is to first look at a map of a sea landscape with a route to follow. The map closes, and the player, which is steering a boat, should remember the map and navigate to find the target location, as previously illustrated on the map. Another task is to remember where you came from, point in that direction, and shoot a projectile back in the direction of the right spot.

### Motor activity, distal temperature, and digital biomarker registration—actigraphy

A research-grade ambulatory wristband (Empatica E4) [71] with sensors that register accelerometric data, peripheral body temperature, electrodermal activity, and photoplethysmography (PPG) [82–84] is applied. PPG employs infrared light to gauge volumetric changes in blood circulation, making it a widely used method for estimating heart rate and obtaining pulse oximetry readings [85, 86]. Heart rate variability analysis displays the function of the autonomic nervous system, and mood disorders are linked with autonomic dysfunction [87]. Autonomic activity can furthermore be assessed by observing electrodermal activity (skin conductance), which measures sweating on the skin, a function regulated by the sympathetic branch of the autonomic nervous system [88]. Accelerometry measures motor activity, with recordings of acceleration in three-dimensional space over time. The information in motor activity is hypothesized to be an objective observation of the inner physiological state [89]. Variations in energy-levels and activation patterns provide information about mood state, as well as circadian rhythmicity, which seems disrupted in depression [90, 91]. Additionally, the peripheral body temperature also expresses circadian rhythmicity [92]. These data will be acquired over a 48-h period to assess the circadian effects of the heart, electrodermal activity, motor activity and body temperature.

### Blood sampling and biobank

Blood samples will be stored in a study-specific biobank at Biobank Haukeland. If the study participants also provide informed consent for prolonged storage of the samples, the material will be transferred to the ECT research biobank (“*Forskningsbiobanken for nevrostimulerende behandling i Helse Vest*”, Regional Comittee for Medical Research Ethics Western Norway (REK West) 2017/925) after the termination of the study. We will collect blood in serum tubes, whole blood in EDTA tubes (Ethylenediaminetetraacetic acid), and PAX-gen tubes (for stabilization of intracellular RNA) for each timepoint. In the automated biobank the serum will be aliquoted in 6 × 260 µl tubes. The EDTA whole blood will be aliquoted and stored in 3 × 2 ml. The samples in PAX-gen tubes will be stored as is.

### Power analysis

Based on a previous study [93], the first hypothesis of this protocol is that baseline global cerebral blood flow (CBF) will separate responders (lower CBF) from nonresponders at a threshold of ~ 45 mL/100 g/min, and that CBF will increase with ECT. The sample size needed to detect a difference at baseline between responders and nonresponders based on the data in Fig. 1 in [93] is 14 per group (1- β = 0.8, α = 0.05). Detection of a treatment-induced change in the regional hippocampal CBF (paired t test) will require a sample of 22 patients. Our sample of 50 patients should be adequate.

Very large sample sizes are needed to boost statistical power and close knowledge gaps (e.g., confirm or refute results from small studies) and to utilize machine-learning tools for outcome prediction. Hence, team science efforts are important, and parts of the analysis will be achieved through data sharing and collaboration with consortia such as the Genomics of ECT International Consortium [94](Gen-ECT-ic) and GEMRIC [77].

## Discussion

The study described in this protocol will provide a comprehensive dataset with opportunity for multiple future analyses that can cast light on the mechanisms of action for ECT and TMS. At the time of submission of this manuscript, the study included 11 ECT patients, 10 TMS patients and 12 healthy controls. We will explore the underlying mechanisms for both the therapeutic and adverse effects of ECT and TMS. Our translational goal is to select the right patients for treatment, also addressing the primary concerns of our patient representatives who ask for personalized recommendations with respect to the likelihood of good outcome versus cognitive side effects. Ultimately, knowledge from the project should also be communicated broadly to inform the public, in order to dispel remaining myths and stigma, surrounding ECT.

### Hypotheses

#### The response to ECT/TMS can be predicted by (low) pretreatment CBF

Neuroimaging studies found abnormal brain functioning in depression [95, 96]. Although several machine-learning algorithms for predicting treatment outcome based on functional [97] or structural [34, 98, 99] neuroimaging have been developed, to date, none of these algorithms have been translated to clinical practice. Hence, a broad approach including novel methods to tackle the prediction challenge is warranted. A promising recent study found that (CBF) before treatment could distinguish responders (lower global CBF) from nonresponders by using ASL-MRI [93]. We hypothesize that responders will have lower pre-ECT global CBF than nonresponders.

By using ASL-MRI, absolute CBF can be estimated without use the of contrast agent – making eventual clinical implementation affordable and free from the risks associated with intravenous injection of gadolinium contrast.

#### The cognitive side-effects of ECT are correlated with treatment-related increases in gray matter

We will investigate the biological mechanisms behind the ECT/TMS response (and side effects) through a combination of imaging, epigenetics, genetics, and proteomics in the framework of the disrupt, potentiate, and rewire (DPR) hypothesis [30]. The mechanisms of ECT are to be understood as a sequence of events where neuronal circuits are disrupted, potentiated, and ultimately rewired. For the TMS patient group, we do not expect the same extent of the disruptive effects but will investigate changes that can explain the efficacy in terms of potentiation and rewiring. The TMS group will also provide a possibility of comparing treatment-related effects across treatment modalities.

### Research questions

In addition to the two primary hypotheses, several analyses can be performed that can shed light on the mechanisms of action suggested by the DPR hypothesis.

#### Neuroimaging

We predict that neuroimaging results in general will be in line with the DPR hypothesis: Disruption will mainly be seen at TP2 and TP3, to a lesser degree at time TP4 and will not be evident at TP5 (Table 1). Signs of potentiation are expected at TP3 and TP4, and rewiring is expected mainly at TP5 (some at TP4). Volume changes at TP3 and TP4 are expected to relate to side effects. We expect some signs of edema indicative of immediate disruption at two hours after the first ECT session, as measured by advanced DWI. Changes in diffusion properties at TP3 and TP4 will reflect potentiation, and changes at follow-up TP5 will reflect rewiring compared to baseline. Resting-state fMRI at follow-up will show connectome stabilization differing from baseline but will be disrupted after the 1st and 6th ECT session.

Based on previous proton magnetic resonance spectroscopy (^1^H-MRS) investigations in ECT, summarized in Erchinger et al. [100], we propose that N-acetylaspartate (a proposed marker of neuronal integrity), will temporarily decrease after ECT, reflecting the disruption in the DPR hypothesis. We also hypothesize that depressed patients will display lower glutamate levels at baseline compared to controls [101] and that an increase in glutamate levels from baseline to after treatment will be associated with positive treatment outcomes for both TMS [102] and ECT patients [103–107].

#### Clinical outcomes

Both ECT and TMS will reduce MADRS scores at TP4, and we expect ECT to be more efficient with a higher proportion of responders and remitters compared to TMS. In patients treated with ECT, we hypothesize a symptom fluctuation following the trajectory of the disruption and symptom load curves [30].

#### Neuropsychological outcomes

We expect group differences between depressed patients and controls at baseline explained by the “state”, “scar”, and "trait" hypotheses [11]. We expect processing speed and attention to improve following remission of depressive symptoms, explained by the “state” hypothesis. Further we expect executive functions to remain relatively stable, as suggested by the “trait” and “scar hypotheses” [11, 108].

Cognitive side effects seen with ECT (disruptive treatment) are not expected to be seen with TMS (nondisruptive treatment). The cognitive tests of spatial navigation will be used to assess specific hippocampal functions, and we expect the results to be in line with the DPR hypothesis, hence a reduction in performance after end treatment (TP4) and normalization at TP5. Moreover, we expect the hippocampal volume change to correlate with a reduction in performance. Reduced spatial navigation performance will also correlate with increased cortical thickness (broadly distributed volume change) for ECT patients. However, improvements relative to baseline are expected (TP5) for hippocampal functions, i.e. functions that are impaired from a “scar perspective”, such as category fluency, memory and language, in line with the concept of the DPR model. Finally, existing differences between the TMS and ECT groups pre- treatment could elucidate on the cognitive profile in treatment resistant MDD and could be explored.

#### Activity monitoring

Patients are expected to show increased daytime activity, decreased variability in activity levels, decreased nighttime activity and improved quality of sleep after ECT treatment [109, 110]. Motor activity will be assessed by both linear and nonlinear analyses of variability and complexity [111]. Furthermore, stabilization of the circadian rhythmicity should be observable in motor activity, as well as in the peripheral body temperature [90, 112]. Sympathetic activation will fluctuate (disrupt and potentiate); however, it will subsequently decrease, alongside an increase in para-sympathetic activation after ECT, observable in digital biomarkers (heart rate, heart rate variability and electrodermal measures) [88]. Finally, a machine learning-based multisensory approach, combining the digital biomarkers and motor activity recordings of Empatica E4, could be an interesting alternative method for analyzing these data [113].

#### Blood biomarkers

Several treatment-response predictors for MDD have been proposed in recent years, but there are currently no blood-based disease progression or therapy-response biomarkers in clinical use [114]. We aim to advance our understanding of the molecular mechanisms underlying both the pathogenesis of severe MDD and the treatment response to ECT by using a multiomics approach on patient blood and serum samples. By using proteomics, genomics, transcriptomics, and epigenetics to generate multiomics profiles of individual patients we hope to identify biological predictors of treatment outcome that can help guiding personalized, interventional strategies.

Previous research has indicated that depression is accompanied by increased levels of inflammatory markers, such as C-reactive protein (CRP) and interleukin-6 [115], which could be particularly relevant for treatment-resistant depression [116]. It has also been suggested that acute ECT sessions transiently increase inflammation, while full treatment series may reduce inflammation [117]. To elucidate the effect of ECT on neuroinflammatory markers and other relevant blood biomarkers, blood samples will be collected at all timepoints. We expect increased levels of neuroinflammatory markers immediately after treatment and in the short term but not at TP4 and TP5 [117].

Large collaborative studies, including the psychiatric genomics consortium (PGC), have recently identified many common genetic risk variants and rare copy number variants in MDD [118]. Treatment-resistant, severe depression is a clinical subgroup that may have a distinct genetic risk profile [119]. We hypothesize that a joint analysis of patients recruited for ECT treatment across the PGC sites will reveal common and rare genetic risk variants. Moreover, we hypothesize that it will also be possible to detect genetic markers of treatment response using this collaborative framework. Clinical data, and data from genome-wide genotyping will be analyzed locally, also shared, and meta-analyzed within international consortia, such as GenECT-ic [94].

Epigenetic markers will be investigated, both to find markers to predict treatment response and markers that change during the treatment.

We expect changes specific to ECT and will test hypotheses from published reports as well as investigate effects related to Brain-Derived Neurotrophic Factor activation, markers of neurogenesis and immunoactivity. As described previously [120], we aim to supplement the measurements of peripheral biomarker levels by peripheral blood messenger ribonucleic acid (mRNA) levels (array-based genome-wide DNA genotyping, methylation profiling of target genes and real-time reverse transcription polymerase chain reaction mRNA measurements) and genome-wide methylation studies.

In summary, the study will provide a comprehensive dataset, that will be used for specific hypothesis testing and explorative investigations to improve our understanding of depression and neurostimulation treatments. The putative biomarkers will be evaluated for inclusion in a future algorithm for the outcome expectancy score. Such a tool could be used for clinical decision support and would provide patients with evidence-based likelihood estimates for clinical outcomes and provide opportunities for more personalized treatment choices.

## Data Availability

All data and blood will be handled according to approvals (ethical committee and data protection authorities) and research regulations at the Haukeland University Hospital (HUH) and at University of Bergen (UiB). Data will be collected and stored on a research server hosted by the IT-department at HUH and at the research server at UiB. Data will be shared with GEMRIC through a secure research server (SAFE) located at the University of Bergen. The dataset for this study is not publicly available due to Norwegian laws and regulations for medical research (Act on Medical and Health Research, General Data Protection Regulation (GDPR) and The Personal Information Act with regulations).

## ﻿Abbreviations

ABCD: Adolescent Brain Cognitive Development—study
AMT: Autobiographical Memory Test
ASL: Arterial Spin Labeling
BDD: Bipolar depressive disorder
BDI: Beck Inventory test
BL: Bilateral
CBF: Cerebral blood flow
CGI: Clinical Global Impression Scale
CRP: C-reactive protein
CWIT: Color word interference test
DLPFC: Dorsolateral prefrontal cortex
DPR: Disrupt, potentiate and rewire
DWI: Diffusion weighted imaging
ECCA: Electroconvulsive Cognitive Assessment
ECT: Electroconvulsive therapy
EDTA: Ethylenediaminetetraacetic acid (edetic acid)
EEG: Electroencephalography
EMQ: Everyday Memory Questionary
EPI: Echo planar imaging
FA: Flip angel
GEMRIC: The Global ECT-MRI Research Collaboration
HUH: Haukeland University Hospital
MADRS: Montgomery and Aasberg Depression Rating Scale
MDD: Major depressive disorder
MMSE: Mini Mental Status
MRI: Magnetic resonance imaging
mRNA: Messenger ribonucleic acid
MRS: Magnetic resonance spectroscopy
NART: National Adult Reading Test
NOK: Norwegian kroner (currency)
PAX-gen tube: Collection of blood for stabilization of intracellular RNA
PDQ-D 5- item: Perceived Deficit Questionary – Depression 5-item
PPG: Photoplethysmography
QIDS-SR: Quick Inventory of Depressive Symptomatology – Self-Report
RAVLT: Rey Auditory Verbal Learning Test
RCFT: Rey – Osterrieth Complex Figure Test
rsfMRI: Resting State Magnetic Resonance Imaging
RUL: Right unilateral
SDMT: Symbol Digit Modality test
TA: Time of acquisition
TE: Echo time
TMS: Transcranial magnetic stimulation
TMT: Trail Making Test
TP: Time point
TPR: Number of persons treated with ECT per 10.000 resident population per year
TR: Repetition time
UiB: University of Bergen
VAMS: Visual Analog Mood Scale

## References

[1] Lepine JP, Briley M (2011). The increasing burden of depression. Neuropsychiatr Dis Treat.

[2] Miller S, Dell’Osso B, Ketter TA (2014). The prevalence and burden of bipolar depression. J Affect Disord.

[3] Diseases GBD, Injuries C (2020). Global burden of 369 diseases and injuries in 204 countries and territories, 1990–2019: a systematic analysis for the Global Burden of Disease Study 2019. Lancet.

[4] Wittchen HU, Jacobi F, Rehm J, Gustavsson A, Svensson M, Jonsson B, Olesen J, Allgulander C, Alonso J, Faravelli C (2011). The size and burden of mental disorders and other disorders of the brain in Europe 2010. Eur Neuropsychopharmacol.

[5] Olesen J, Gustavsson A, Svensson M, Wittchen HU, Jonsson B (2012). group Cs, European Brain C: the economic cost of brain disorders in Europe. Eur J Neurol.

[6] Sinyor M, Schaffer A, Levitt A (2010). The sequenced treatment alternatives to relieve depression (STAR*D) trial: a review. Can J Psychiatry.

[7] Semkovska M, Quinlivan L, O'Grady T, Johnson R, Collins A, O'Connor J, Knittle H, Ahern E, Gload T (2019). Cognitive function following a major depressive episode: a systematic review and meta-analysis. Lancet Psychiatry.

[8] Hasselbalch BJ, Knorr U, Kessing LV (2011). Cognitive impairment in the remitted state of unipolar depressive disorder: a systematic review. J Affect Disord.

[9] Hammar Å, Ardal G (2009). Cognitive functioning in major depression–a summary. Front Hum Neurosci.

[10] Allott K, Fisher CA, Amminger GP, Goodall J, Hetrick S (2016). Characterizing neurocognitive impairment in young people with major depression: state, trait, or scar?. Brain Behav.

[11] Hammar Å, Ronold EH, Rekkedal GA (2022). Cognitive impairment and neurocognitive profiles in major depression-a clinical perspective. Front Psychiatry.

[12] Gorwood P, Corruble E, Falissard B, Goodwin GM (2008). Toxic effects of depression on brain function: impairment of delayed recall and the cumulative length of depressive disorder in a large sample of depressed outpatients. Am J Psychiatry.

[13] Moylan S, Maes M, Wray NR, Berk M (2013). The neuroprogressive nature of major depressive disorder: pathways to disease evolution and resistance, and therapeutic implications. Mol Psychiatry.

[14] Carney S, Cowen P, Dearness K, Eastaugh J (2003). Efficacy and safety of electroconvulsive therapy in depressive disorders: a systematic review and meta-analysis. Lancet.

[15] Schoeyen HK, Kessler U, Andreassen OA, Auestad BH, Bergsholm P, Malt UF, Morken G, Oedegaard KJ, Vaaler A (2015). Treatment-resistant bipolar depression: a randomized controlled trial of electroconvulsive therapy versus algorithm-based pharmacological treatment. Am J Psychiatry.

[16] Voineskos D, Daskalakis ZJ, Blumberger DM (2020). Management of treatment-resistant depression: challenges and strategies. Neuropsychiatr Dis Treat.

[17] Aoki Y, Yamaguchi S, Ando S, Sasaki N, Bernick PJ, Akiyama T (2016). The experience of electroconvulsive therapy and its impact on associated stigma: a meta-analysis. Int J Soc Psychiatry.

[18] Read J, Cunliffe S, Jauhar S, McLoughlin DM (2019). Should we stop using electroconvulsive therapy?. BMJ.

[19] Peterchev AV, Rosa MA, Deng ZD, Prudic J, Lisanby SH (2010). Electroconvulsive therapy stimulus parameters: rethinking dosage. J ECT.

[20] Leiknes KA, Jarosh-von Schweder L, Hoie B (2012). Contemporary use and practice of electroconvulsive therapy worldwide. Brain Behav.

[21] Fraser LM, O'Carroll RE, Ebmeier KP (2008). The effect of electroconvulsive therapy on autobiographical memory: a systematic review. J ECT.

[22] Sackeim HA, Ross FR, Hopkins N, Calev L, Devanand DP (1987). Subjective side effects acutely following ECT: associations with treatment modality and clinical response. Convuls Ther.

[23] Semkovska M, Knittle H, Leahy J, Rasmussen JR. Subjective cognitive complaints and subjective cognition following electroconvulsive therapy for depression: a systematic review and meta-analysis. Aust N Z J Psychiatry. 2023;57(1):21–33. 10.1177/00048674221089231 35362328

[24] Landry M, Moreno A, Patry S, Potvin S, Lemasson M (2021). Current practices of electroconvulsive therapy in mental disorders: a systematic review and meta-analysis of short and long-term cognitive effects. J ECT.

[25] Semkovska M, McLoughlin DM (2010). Objective cognitive performance associated with electroconvulsive therapy for depression: a systematic review and meta-analysis. Biol Psychiatry.

[26] Kessler U, Schoeyen HK, Andreassen OA, Eide GE, Malt UF, Oedegaard KJ, Morken G, Sundet K, Vaaler AE (2014). The effect of electroconvulsive therapy on neurocognitive function in treatment-resistant bipolar disorder depression. J Clin Psychiatry.

[27] Kirov GG, Owen L, Ballard H, Leighton A, Hannigan K, Llewellyn D, Escott-Price V, Atkins M (2016). Evaluation of cumulative cognitive deficits from electroconvulsive therapy. Br J Psychiatry.

[28] Semkovska M, McLoughlin DM (2013). Measuring retrograde autobiographical amnesia following electroconvulsive therapy: historical perspective and current issues. J ECT.

[29] Rasmussen KG (2016). What type of cognitive testing should be part of routine electroconvulsive therapy practice?. J ECT.

[30] Ousdal OT, Brancati GE, Kessler U, Erchinger V, Dale AM, Abbott C, Oltedal L (2022). The neurobiological effects of electroconvulsive therapy studied through magnetic resonance: what have we learned, and where do we go?. Biol Psychiatry.

[31] Nordanskog P, Larsson MR, Larsson EM, Johanson A (2014). Hippocampal volume in relation to clinical and cognitive outcome after electroconvulsive therapy in depression. Acta Psychiatr Scand.

[32] Oltedal L, Narr KL, Abbott C, Anand A, Argyelan M, Bartsch H, Dannlowski U, Dols A, van Eijndhoven P, Emsell L (2018). Volume of the human hippocampus and clinical response following electroconvulsive therapy. Biol Psychiatry.

[33] Argyelan M, Oltedal L, Deng Z-D, Wade B, Bikson M, Joanlanne A, Sanghani S, Bartsch H, Cano M, Dale AM (2019). Electric field causes volumetric changes in the human brain. eLife.

[34] Mulders PCR, Llera A, Beckmann CF, Vandenbulcke M, Stek M, Sienaert P, Redlich R, Petrides G, Oudega ML, Oltedal L (2020). Structural changes induced by electroconvulsive therapy are associated with clinical outcome. Brain Stimul.

[35] Deng ZD, Argyelan M, Miller J, Quinn DK, Lloyd M, Jones TR, Upston J, Erhardt E, McClintock SM, Abbott CC (2022). Electroconvulsive therapy, electric field, neuroplasticity, and clinical outcomes. Mol Psychiatry.

[36] van Oostrom I, van Eijndhoven P, Butterbrod E, van Beek MH, Janzing J, Donders R, Schene A, Tendolkar I (2018). Decreased cognitive functioning after electroconvulsive therapy is related to increased hippocampal volume: exploring the role of brain plasticity. J ECT.

[37] Laroy M, Bouckaert F, Vansteelandt K, Obbels J, Dols A, Emsell L, Stek M, Vandenbulcke M, Sienaert P (2019). Association between hippocampal volume change and change in memory following electroconvulsive therapy in late-life depression. Acta Psychiatr Scand.

[38] Cowey A (2005). The Ferrier Lecture 2004 what can transcranial magnetic stimulation tell us about how the brain works?. Philos Trans R Soc Lond B Biol Sci.

[39] Walsh V, Cowey A (2000). Transcranial magnetic stimulation and cognitive neuroscience. Nat Rev Neurosci.

[40] De Raedt R, Vanderhasselt MA, Baeken C (2015). Neurostimulation as an intervention for treatment resistant depression: From research on mechanisms towards targeted neurocognitive strategies. Clin Psychol Rev.

[41] O'Reardon JP, Solvason HB, Janicak PG, Sampson S, Isenberg KE, Nahas Z, McDonald WM, Avery D, Fitzgerald PB, Loo C (2007). Efficacy and safety of transcranial magnetic stimulation in the acute treatment of major depression: a multisite randomized controlled trial. Biol Psychiatry.

[42] George MS, Wassermann EM, Williams WA, Callahan A, Ketter TA, Basser P, Hallett M, Post RM (1995). Daily repetitive transcranial magnetic stimulation (rTMS) improves mood in depression. NeuroReport.

[43] Iimori T, Nakajima S, Miyazaki T, Tarumi R, Ogyu K, Wada M, Tsugawa S, Masuda F, Daskalakis ZJ, Blumberger DM (2019). Effectiveness of the prefrontal repetitive transcranial magnetic stimulation on cognitive profiles in depression, schizophrenia, and Alzheimer's disease: a systematic review. Prog Neuropsychopharmacol Biol Psychiatry.

[44] McClintock SM, Reti IM, Carpenter LL, McDonald WM, Dubin M, Taylor SF, et al. Consensus Recommendations for the Clinical Application of Repetitive Transcranial Magnetic Stimulation (rTMS) in the Treatment of Depression. J Clin Psychiatry. 2018;79(1):35–48. 10.4088/JCP.16cs10905 PMC5846193 28541649

[45] Lefaucheur JP, Aleman A, Baeken C, Benninger DH, Brunelin J, Di Lazzaro V, Filipovic SR, Grefkes C, Hasan A, Hummel FC (2020). Evidence-based guidelines on the therapeutic use of repetitive transcranial magnetic stimulation (rTMS): An update (2014–2018). Clin Neurophysiol.

[46] Baeken C, Brem AK, Arns M, Brunoni AR, Filipcic I, Ganho-Avila A, Langguth B, Padberg F, Poulet E, Rachid F (2019). Repetitive transcranial magnetic stimulation treatment for depressive disorders: current knowledge and future directions. Curr Opin Psychiatry.

[47] Montgomery SA, Åsberg M (1979). A new depression scale designed to be sensitive to change. Br J Psychiatry.

[48] Beck AT, Ward CH, Mendelson M, Mock J, Erbaugh J (1961). An Inventory for Measuring Depression. Arch Gen Psychiatry.

[49] Busner J, Targum SD (2007). The clinical global impressions scale: applying a research tool in clinical practice. Psychiatry (Edgmont (Pa : Township)).

[50] Sunderland A, Harris JE, Baddeley AD (1983). Do laboratory tests predict everyday memory? A neuropsychological study. J Verbal Learn Verbal Behav.

[51] Strobel C, Engedal K (2018). Norsk revidert mini mental status evaluering (MMSE-N3). Aldring og helse.

[52] Rey A: L'examen psychologique dans les cas d'encéphalopathie traumatique.(Les problems.). Archives de psychologie 1941.

[53] Schoenberg MR, Dawson KA, Duff K, Patton D, Scott JG, Adams RL (2006). Test performance and classification statistics for the Rey Auditory Verbal Learning Test in selected clinical samples. Arch Clin Neuropsychol.

[54] Fine EM, Delis DC, Kreutzer JS, DeLuca J, Caplan B (2011). Delis–Kaplan Executive Functioning System. Encyclopedia of Clinical Neuropsychology.

[55] Wechsler D: WAIS-IV. In: Encyclopedia of Clinical Neuropsychology*.* edn. Edited by Kreutzer JS, DeLuca J, Caplan B. New York: Springer New York; 2011: 2667–2667.

[56] Williams JM, Broadbent K (1986). Autobiographical memory in suicide attempters. J Abnorm Psychol.

[57] van Walsem M, Tyvoll H (2012). Montreal cognitive assessment (MoCA). Norwegian version.

[58] Nelson HE (1982). National Adult Reading Test (NART) Test Manual.

[59] Rush AJ, Trivedi MH, Ibrahim HM, Carmody TJ, Arnow B, Klein DN, Markowitz JC, Ninan PT, Kornstein S, Manber R (2003). The 16-Item Quick Inventory of Depressive Symptomatology (QIDS), clinician rating (QIDS-C), and self-report (QIDS-SR): a psychometric evaluation in patients with chronic major depression. Biol Psychiatry.

[60] Killgore WDS (1999). The visual analogue mood scale: can a single-item scale accurately classify depressive mood state?. Psychol Rep.

[61] Doeller CF, Barry C, Burgess N (2010). Evidence for grid cells in a human memory network. Nature.

[62] Doeller CF, King JA, Burgess N (2008). Parallel striatal and hippocampal systems for landmarks and boundaries in spatial memory. Proc Natl Acad Sci U S A.

[63] Hartley T, Bird CM, Chan D, Cipolotti L, Husain M, Vargha-Khadem F, Burgess N (2007). The hippocampus is required for short-term topographical memory in humans. Hippocampus.

[64] Coutrot A, Silva R, Manley E, de Cothi W, Sami S, Bohbot VD, Wiener JM, Holscher C, Dalton RC, Hornberger M (2018). Global Determinants of Navigation Ability. Curr Biol.

[65] Osterrieth PA: Le Test de copie d'une figure complexe : contribution à l'etude de la perception et de la mémoire. Delachaux & Niestlé; 1944.

[66] Sullivan MJ, Edgley K, Dehoux E. A survey of multiple sclerosis: I. Perceived cognitive problems and compensatory strategy use. Can J Rehabil. 1990;4(2):99–105.

[67] Sumiyoshi T, Uchida H, Watanabe K, Oosawa M, Ren H, Moriguchi Y, Fujikawa K, Fernandez J (2022). Validation and functional relevance of the short form of the perceived deficits questionnaire for depression for japanese patients with major depressive disorder. Neuropsychiatr Dis Treat.

[68] Hermida AP, Goldstein FC, Loring DW, McClintock SM, Weiner RD, Reti IM, Janjua AU, Ye Z, Peng L, Tang Y-I (2020). ElectroConvulsive therapy Cognitive Assessment (ECCA) tool: A new instrument to monitor cognitive function in patients undergoing ECT. J Affect Disord.

[69] Hugdahl K, Westerhausen R, Alho K, Medvedev S, Laine M, Hamalainen H (2009). Attention and cognitive control: unfolding the dichotic listening story. Scand J Psychol.

[70] Bless J, Westerhausen R, Arciuli J, Kompus K, Gudmundsen M, Hugdahl K. “Right on all Occasions?” – On the Feasibility of Laterality Research Using a Smartphone Dichotic Listening Application. Front Psychol. 2013;4(42):1–10. 10.3389/fpsyg.2013.00042 PMC3566356 23404376

[71] Empatica.com [https://www.empatica.com/en-int/]

[72] d'Elia G (1970). Unilateral electroconvulsive therapy. Acta Psychiatr Scand.

[73] Kessler U, Vaaler AE, Schoyen H, Oedegaard KJ, Bergsholm P, Andreassen OA, Malt UF, Morken G (2010). The study protocol of the Norwegian randomized controlled trial of electroconvulsive therapy in treatment resistant depression in bipolar disorder. BMC Psychiatry.

[74] Casey BJ, Cannonier T, Conley MI, Cohen AO, Barch DM, Heitzeg MM, Soules ME, Teslovich T, Dellarco DV, Garavan H (2018). The Adolescent Brain Cognitive Development (ABCD) study: Imaging acquisition across 21 sites. Dev Cogn Neurosci.

[75] Hagler DJ, Hatton SN, Makowski C, Cornejo MD, Fair DA, Dick AS, et al. Image processing and analysis methods for the Adolescent Brain Cognitive Development Study. Neuroimage. 2019;202:1–39. 10.1016/j.neuroimage.2019.116091 PMC6981278 31415884

[76] Wilkinson ST, Sanacora G, Bloch MH (2017). Hippocampal volume changes following electroconvulsive therapy: a systematic review and meta-analysis. Biol Psychiatry Cogn Neurosci Neuroimaging.

[77] Oltedal L, Bartsch H, Sørhaug OJE, Kessler U, Abbott C, Dols A, Stek ML, Ersland L, Emsell L, van Eijndhoven P (2017). The Global ECT-MRI Research Collaboration (GEMRIC): Establishing a multi-site investigation of the neural mechanisms underlying response to electroconvulsive therapy. NeuroImage.

[78] Robinson JA (1976). Sampling autobiographical memory. Cogn Psychol.

[79] Ronold EH, Joormann J, Hammar A (2022). Computerized working memory training in remission from major depressive disorder: effects on emotional working memory, processing speed, executive functions, and associations with symptoms. Front Behav Neurosci.

[80] Bless JJ, Westerhausen R, von Koss TJ, Gudmundsen M, Kompus K, Hugdahl K (2015). Laterality across languages: Results from a global dichotic listening study using a smartphone application. Laterality.

[81] Lee SM, Shin J, Lee I. Significance of visual scene-based learning in the hippocampal systems across mammalian species. Hippocampus. 2023;33:505–21. 10.1002/hipo.23483 36458555

[82] Schuurmans AAT, de Looff P, Nijhof KS, Rosada C, Scholte RHJ, Popma A, Otten R (2020). Validity of the Empatica E4 Wristband to Measure Heart Rate Variability (HRV) Parameters: a Comparison to Electrocardiography (ECG). J Med Syst.

[83] Milstein N, Gordon I (2020). Validating measures of electrodermal activity and heart rate variability derived from the empatica E4 utilized in research settings that involve interactive dyadic states. Front Behav Neurosci.

[84] Menghini L, Gianfranchi E, Cellini N, Patron E, Tagliabue M, Sarlo M (2019). Stressing the accuracy: wrist-worn wearable sensor validation over different conditions. Psychophysiology.

[85] Castaneda D, Esparza A, Ghamari M, Soltanpur C, Nazeran H (2018). A review on wearable photoplethysmography sensors and their potential future applications in health care. Int J Biosens Bioelectron.

[86] Stautland A, Jakobsen P, Fasmer OB, Osnes B, Torresen J, Nordgreen T, et al. Reduced heart rate variability during mania in a repeated naturalistic observational study. Front Psychiatry. 2023;14:1–8. 10.3389/fpsyt.2023.1250925 PMC10513449 37743991

[87] Bassett D (2016). A literature review of heart rate variability in depressive and bipolar disorders. Aust N Z J Psychiatry.

[88] Huang WL, Ko LC, Liao SC (2022). The association between heart rate variability and skin conductance: a correlation analysis in healthy individuals and patients with somatic symptom disorder comorbid with depression and anxiety. J Int Med Res.

[89] Scott J, Murray G, Henry C, Morken G, Scott E, Angst J, Merikangas KR, Hickie IB (2017). Activation in bipolar disorders: a systematic review. JAMA Psychiat.

[90] Murray G, Gottlieb J, Hidalgo MP, Etain B, Ritter P, Skene DJ, Garbazza C, Bullock B, Merikangas K, Zipunnikov V (2020). Measuring circadian function in bipolar disorders: Empirical and conceptual review of physiological, actigraphic, and self-report approaches. Bipolar Disord.

[91] McCarthy MJ, Gottlieb JF, Gonzalez R, McClung CA, Alloy LB, Cain S, Dulcis D, Etain B, Frey BN, Garbazza C (2022). Neurobiological and behavioral mechanisms of circadian rhythm disruption in bipolar disorder: A critical multi-disciplinary literature review and agenda for future research from the ISBD task force on chronobiology. Bipolar Disord.

[92] Refinetti R (2020). Circadian rhythmicity of body temperature and metabolism. Temperature (Austin).

[93] Leaver AM, Vasavada M, Joshi SH, Wade B, Woods RP, Espinoza R, Narr KL (2019). Mechanisms of antidepressant response to electroconvulsive therapy studied with perfusion magnetic resonance imaging. Biol Psychiatry.

[94] Baune BT, Soda T, Gen ECTi, Sullivan PF, Zandi P (2019). The Genomics of Electroconvulsive Therapy International Consortium (GenECT-ic). Lancet Psychiatry.

[95] Goodman ZT, Bainter SA, Kornfeld S, Chang C, Nomi JS, Uddin LQ (2021). Whole-brain functional dynamics track depressive symptom severity. Cereb Cortex.

[96] Hamilton JP, Furman DJ, Chang C, Thomason ME, Dennis E, Gotlib IH (2011). Default-mode and task-positive network activity in major depressive disorder: implications for adaptive and maladaptive rumination. Biol Psychiatry.

[97] van Waarde JA, Scholte HS, van Oudheusden LJ, Verwey B, Denys D, van Wingen GA (2015). A functional MRI marker may predict the outcome of electroconvulsive therapy in severe and treatment-resistant depression. Mol Psychiatry.

[98] Redlich R, Opel N, Grotegerd D, Dohm K, Zaremba D, Burger C, Munker S, Muhlmann L, Wahl P, Heindel W (2016). Prediction of individual response to electroconvulsive therapy via machine learning on structural magnetic resonance imaging data. JAMA Psychiat.

[99] Jiang R, Abbott CC, Jiang T, Du Y, Espinoza R, Narr KL, Wade B, Yu Q, Song M, Lin D (2017). SMRI biomarkers predict electroconvulsive treatment outcomes: accuracy with independent data sets. Neuropsychopharmacology.

[100] Erchinger VJ, Ersland L, Aukland SM, Abbott CC, Oltedal L (2021). Magnetic resonance spectroscopy in depressed subjects treated with electroconvulsive therapy-a systematic review of literature. Front Psychiatry.

[101] Yildiz-Yesiloglu A, Ankerst DP (2006). Review of 1H magnetic resonance spectroscopy findings in major depressive disorder: a meta-analysis. Psychiatry Res.

[102] Gonsalves MA, White TL, Barredo J, Fukuda AM, Joyce HE, Harris AD, Carpenter LL (2022). Repetitive transcranial magnetic stimulation-associated changes in neocortical metabolites in major depression: a systematic review. Neuroimage Clin.

[103] Michael N, Erfurth A, Ohrmann P, Arolt V, Heindel W, Pfleiderer B (2003). Metabolic changes within the left dorsolateral prefrontal cortex occurring with electroconvulsive therapy in patients with treatment resistant unipolar depression. Psychol Med.

[104] Michael N, Erfurth A, Ohrmann P, Arolt V, Heindel W, Pfleiderer B (2003). Neurotrophic effects of electroconvulsive therapy: a proton magnetic resonance study of the left amygdalar region in patients with treatment-resistant depression. Neuropsychopharmacology.

[105] Njau S, Joshi SH, Espinoza R, Leaver AM, Vasavada M, Marquina A, Woods RP, Narr KL (2017). Neurochemical correlates of rapid treatment response to electroconvulsive therapy in patients with major depression. J Psychiatry Neurosci.

[106] Pfleiderer B, Michael N, Erfurth A, Ohrmann P, Hohmann U, Wolgast M, Fiebich M, Arolt V, Heindel W (2003). Effective electroconvulsive therapy reverses glutamate/glutamine deficit in the left anterior cingulum of unipolar depressed patients. Psychiatry Res.

[107] Zhang J, Narr KL, Woods RP, Phillips OR, Alger JR, Espinoza RT (2013). Glutamate normalization with ECT treatment response in major depression. Mol Psychiatry.

[108] Ronold EH, Schmid MT, Oedegaard KJ, Hammar A (2020). A Longitudinal 5-year follow-up study of cognitive function after first episode major depressive disorder: exploring state scar and trait effects. Front Psychiatry.

[109] Burton C, McKinstry B, Szentagotai Tatar A, Serrano-Blanco A, Pagliari C, Wolters M (2013). Activity monitoring in patients with depression: a systematic review. J Affect Disord.

[110] Jakobsen P, Garcia-Ceja E, Riegler M, Stabell LA, Nordgreen T, Torresen J, Fasmer OB, Oedegaard KJ (2020). Applying machine learning in motor activity time series of depressed bipolar and unipolar patients compared to healthy controls. PLoS One.

[111] Jakobsen P, Stautland A, Riegler MA, Cote-Allard U, Sepasdar Z, Nordgreen T, Torresen J, Fasmer OB, Oedegaard KJ (2022). Complexity and variability analyses of motor activity distinguish mood states in bipolar disorder. PLoS One.

[112] Cuesta M, Boudreau P, Cermakian N, Boivin DB (2017). Skin temperature rhythms in humans respond to changes in the timing of sleep and light. J Biol Rhythms.

[113] Cote-Allard U, Jakobsen P, Stautland A, Nordgreen T, Fasmer OB, Oedegaard KJ, et al. Long-Short Ensemble Network for Bipolar Manic-Euthymic State Recognition Based on Wrist-worn Sensors. arXiv. 2022;2107.00710v3:1–12.

[114] Kennis M, Gerritsen L, van Dalen M, Williams A, Cuijpers P, Bockting C (2020). Prospective biomarkers of major depressive disorder: a systematic review and meta-analysis. Mol Psychiatry.

[115] Smith KJ, Au B, Ollis L, Schmitz N (2018). The association between C-reactive protein, Interleukin-6 and depression among older adults in the community: a systematic review and meta-analysis. Exp Gerontol.

[116] Lullau APM, Haga EMW, Ronold EH, Dwyer GE (2023). Antidepressant mechanisms of ketamine: a review of actions with relevance to treatment-resistance and neuroprogression. Front Neurosci.

[117] Yrondi A, Sporer M, Peran P, Schmitt L, Arbus C, Sauvaget A (2018). Electroconvulsive therapy, depression, the immune system and inflammation: a systematic review. Brain Stimul.

[118] Howard DM, Adams MJ, Clarke TK, Hafferty JD, Gibson J, Shirali M, Coleman JRI, Hagenaars SP, Ward J, Wigmore EM (2019). Genome-wide meta-analysis of depression identifies 102 independent variants and highlights the importance of the prefrontal brain regions. Nat Neurosci.

[119] Cai N, Revez JA, Adams MJ, Andlauer TFM, Breen G, Byrne EM, Clarke TK, Forstner AJ, Grabe HJ, Hamilton SP (2020). Minimal phenotyping yields genome-wide association signals of low specificity for major depression. Nat Genet.

[120] Oltedal L, Kessler U, Ersland L, Grüner R, Andreassen OA, Haavik J, Hoff PI, Hammar Å, Dale AM, Hugdahl K (2015). Effects of ECT in treatment of depression: study protocol for a prospective neuroradiological study of acute and longitudinal effects on brain structure and function. BMC Psychiatry.

